# Cellular Immunity in Chronic Kidney Disease and Changes After Kidney Transplantation

**DOI:** 10.3389/ti.2026.15622

**Published:** 2026-02-03

**Authors:** Georgios Lioulios, Eleni Moisidou, Michalis Christodoulou, Efstratios Kasimatis, Aliki Xochelli, Evaggelos Memmos, Stamatia Stai, Maria Stangou, Asimina Fylaktou

**Affiliations:** 1 Department of Nephrology, 424 General Military Hospital, Thessaloniki, Greece; 2 First Department of Nephrology, Aristotle University of Thessaloniki, General Hospital of Thessaloniki “Hippokratio”, Thessaloniki, Greece; 3 National Peripheral Histocompatibility Center, Department of Immunology, General Hospital of Thessaloniki “Hippokratio”, Thessaloniki, Greece; 4 Department of Nephrology, “Papageorgiou” General Hospital, Thessaloniki, Greece

**Keywords:** CD28^−^ T cells, chronic kidney disease, dialysis, senescence, T cells, transplantation, regulatory T cells, natural killers

## Abstract

This study evaluates changes in cellular immunity components, from chronic kidney disease stage V (CKD-V) to long-term transplantation (lKTx). We applied flow cytometry to determine total, CD4^+^, CD8^+^, CD28^−^ T-lymphocytes, Natural killer cells (NK) and regulatory T-lymphocytes (Tregs), in peripheral blood of 56 patients with CKD-V, 207 patients on hemodialysis (HD), 149 recently transplanted (rKTx), 26 lKTx patients and 49 healthy volunteers as a control group (CG). Lymphocyte proportion decreased in CKD vs. CG [20.2 (14.4–25.8) vs. 29.7 (24.4–38.1)%, p < 0.001] without further deterioration in HD [19.9 (15.3–23.7)%, p = 0.83 vs. CKD-V] and increased in rKTx [24.7 (20–32.6)%, p < 0.001 vs. HD], and lKTx [25.5 (21.9–35)%, p = 0.16 vs. CG]. Similar kinetics were observed in CD4^+^ subpopulations, however CD8^+^ T-cells gradually increased from CG to lKTx. NK remained stable in CKD-V and HD, reduced in rKTx, and marginally increased in lKTx. Tregs gradually declined until HD and suboptimally improved with transplantation. CD28^−^ subpopulations largely increased in lKTx, compared with HD [CD4^+^CD28^−^: 12.6 (4.7–27.6) vs. 6 (2.1–13.2)%, p = 0.006, CD8^+^CD28^−^: 68.4 (54.4–90.3) vs. 45.5 (28.4–58.9)%, p < 0.001], independently of age for CD8^+^CD28^−^ (p = 0.33). Cellular immunity subpopulations show significant changes in the spectrum of CKD, with transplantation restoring total lymphocytes and CD4^+^ T-cells, but not Tregs and NK. LKTx was associated with a large increase in CD28^−^ subpopulations.

## Graphical Abstract

**Figure:**
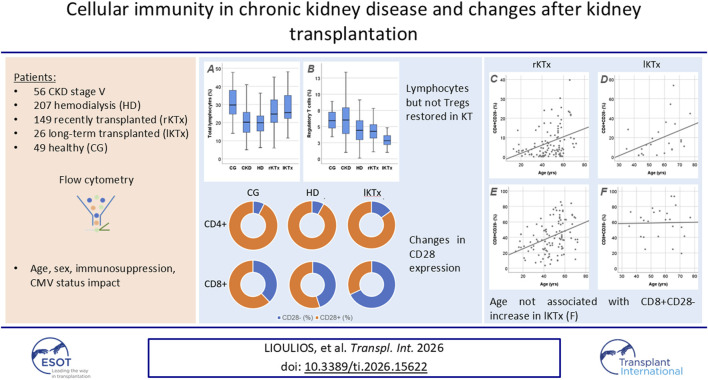

## Introduction

Chronic kidney disease (CKD) is characterized by phenotypic and functional alterations of cellular elements of immune system, leading to relative immunodeficiency in these patients. This is clinically expressed as increased susceptibility in infections, decreased response to vaccination, as well as increased risk for neoplasia, potentially due to altered immune surveillance [1–3]. As a result, patients in hemodialysis (HD) have a greater mortality rate, with life expectancy being as low as 25%–30% compared to healthy individuals of the same age [1].

CKD has an impact on all cellular populations of acquired immunity, with T cells being the most well studied. One main alteration is the loss of CD28 expression on their surface [4]. CD28 is a transmembrane protein that provides the second signal in the immune synapse between T cell and antigen-presenting cell and thus is indispensable for T cell activation [5]. Failure of CD28 to bind to its ligands CD80 and CD86, during T cell receptor recognition of antigen presented on HLA molecule, leads to impaired of T cell activation and anergy [6]. Moreover, other lymphocytic populations such as regulatory T cells (Tregs) and natural killers (NK) have been described to be altered in CKD patients [7].

The substitution of kidney function with kidney transplantation improves immune function, potentially partially restoring the above-described cellular alterations. However, these alterations have not been adequately described in the whole cohort of CKD (pre-HD, HD, short- and long-term kidney transplantation). The purpose of the present study was to evaluate changes in cellular immunity elements from non-dialysis stage V CKD to long lasting kidney transplantation.

## Patients and Methods

### Study Design

This is an observational study conducted in the First Department of Nephrology of Aristotle University of Thessaloniki, in collaboration with the Department of Immunology, National Histocompatibility Center, of General Hospital of Thessaloniki “Hippokration”, from June 2021 to June 2024. Four groups of patients were included in the study; patients with CKD stage V not yet in dialysis (estimated glomerular filtration rate - eGFR: <15 mL/min/1.73 m^2^) – CKD-V group; patients in chronic maintenance hemodialysis for at least 1 year – HD group; recently transplanted patients (1 year since kidney transplantation) – rKTx group; long transplanted patients (>17 years since kidney transplantation) – lKTx group. The cut-off of 17 years was the average predicted 3rd quantile of living and deceased donors graft survival in years, as reported in a large study published in 2021 [8]. All transplanted patients had received an ABO and HLA compatible graft, with no detectable anti-HLA antibodies against donor’s HLA molecules.

Demographic, clinical and laboratory characteristics, medication, and also, hemodialysis prescription data (for those on HD), were recorded on the day of enrollment. For all patients IgG antibodies levels against cytomegalovirus were measured. Patients eligible for the study were Caucasians, adults, with a minimum of 12-month follow-up in our department. All patients should be in stable treatment for the last 3 months, with well controlled hypeparathyroidism and anemia. Patients undergoing hemodialysis were on a chronic maintenance thrice a week program with Kt/V >1.2, with satisfactory regulation of biochemical parameters, according to international guidelines. For rKTx and lKTx patients, eGFR should be >30 mL/min/1.73 m^2^ calculated using the equation CKD-EPI 2021 for creatinine. Patients were excluded in case of a recent infection or vaccination (<3 months), active malignancy, autoimmune, or hematologic disorder (<5 years), or prior immunosuppressive therapy within the past year (excluding steroids, MMF, and cyclosporine or tacrolimus for transplant patients). Moreover, we excluded transplanted patients receiving agents other than those mentioned in the following section, such as everolimus or belatacept, or patients on a corticosteroid sparing scheme. Forty-nine age- and sex-matched healthy volunteers were used as a control group (CG). In all patient groups and the CG, we assessed immune phenotype with flow cytometry in peripheral blood samples, as described in the relevant section.

### Treatment Protocols

All transplanted patients had received standardized immunosuppressive treatment according to the Transplant Clinic protocol. Induction therapy consisted of basiliximab or anti-thymocyte globulin (ATG), according to the following. The standard induction regimen consisted of two doses of basiliximab 20 mg (administered 60 min before and on day 4 post-transplant) and two doses of methylprednisolone 500 mg, 12 and 1 h before transplantation. Patients in medium immunological risk, for example patients with history of donor specific antibodies (DSA) positivity, high expected cold ischemia time or marginal donor, received induction therapy with 1.5 mg/kg ATG for 4 days to a total dose of 6 mg/kg. Maintenance therapy included methylprednisolone (125 mg intravenously on day one and then 16 mg orally until day 14, with a gradual tapering until day 42), tacrolimus (plasma levels 6–8 ng/mL for the first year), and mycophenolate mofetil (2 g per day until day 14 and then 1 g daily). Patients with T-cell mediated rejection episodes received ATG, whereas patients with antibody mediated rejection episodes received corticosteroids and/or plasma exchange, depending on the indication.

All study participants signed an informed consent prior to enrollment. The study was conducted in accordance with the Declaration of Helsinki and was approved by the Bioethics Committee of the Aristotle University of Thessaloniki (no. 2348/24-11-2019)

### Flow Cytometry

**FIGURE 1:**
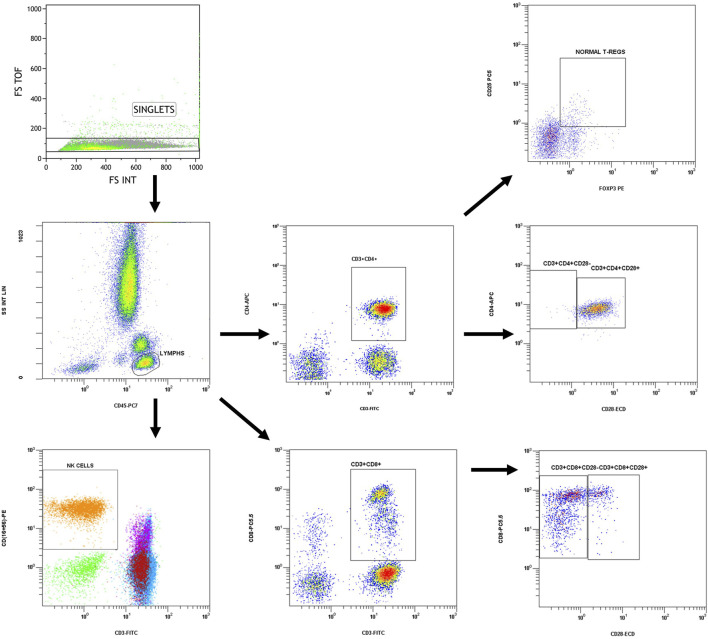
Gating strategy.

Immune phenotype examination was performed with flow cytometry on whole blood samples collected in K2EDTA tubes, within a short period after collection and in any case in no more than 12 h. Analysis was performed on a Navios EX Flow Cytometer (Beckman Coulter), according to the manufacturer’s instructions, using the following conjugated antibodies: CD45 PC7 (Clone J33), CD3 FITC (Clone UCHT1), CD16‐CD56 PE [CD16: Clone 3G8, CD56: Clone N901(NKH-1)], CD4 APC (Clone 13B8.2), CD8 PC5.5 (Clone B9.11), CD4 FITC (Clone SFCI12T4D11), CD25 PC5 (Clone B1.49.9), and FOXP3 PE (Clone 259D, intracellular staining). Percentages of CD4^+^ (CD3^+^CD4^+^), CD8^+^ (CD3^+^CD8^+^), regulatory (CD4^+^CD25^+^FOXP3^+^) T lymphocytes as well as NK cells (CD3^−^CD16^+^CD56^+^) and CD28^−^ (CD4^+^CD28^−^ and CD8^+^CD8^−^) T cells were determined. The total lymphocyte count was also determined from a blood count in the same sample. Gating strategy is shown in Figure 1 and was manually adjusted in each case.

Singlets discrimination was performed with plotting forward scatter integral (FS-INT) versus forward scatter Time of Flight (FS-TOF). Events deviating from the singlet line were considered cell aggregates and discarded from analysis. Compensation settings were determined before experimental analysis, by using single stained control samples. One sample for each conjugated antibody was analyzed separately to measure the spectral overlap of each fluorochrome’s emission into other detectors and mathematical compensation was applied and adjusted manually to correct the spillover. No viability check was needed, as the analysis was performed in fresh blood samples.

### Statistical Analysis

Statistical analysis was performed with Statistical Package for the Social Sciences (SPSS) for Windows, version 25 (IBM, NY, United States). Continuous variables are presented as median (25th – 75th percentile). Quantitative variables were compared with Mann-Whitney U-test between two groups, or with Kruskal-Wallis H-test among multiple groups. The χ^2^ test was used to compare differences in non-quantitative variables between two groups. Correlation between two continuous variables was assessed using the Spearman test. The result was considered significant when R was greater than the critical value for the degrees of freedom. Linear univariate regression was used to predict simple models after logarithmic transformation of the data, and linear multivariate analysis was applied to find independent factors associated with a variable. Statistical significance was considered at a p value of <0.05. Finally, in cases of comparison of more than two different populations, the Bonferroni correction was applied.

Given the use of non-parametric tests between CG, HD and lKTx patients and for a significance level of 0.005 (after Bonferroni correction) with 80% power, the expected effect size for the variable “CD8^+^CD28^−^ T cells percentage” was estimated based on a previous similar study [9], corresponding to a moderate effect (Cohen’s d ≈ 0.48, corresponding to r ≈ 0.23). Based on this effect size, the adequate sample size for the lKTx group was calculated to N = 121 patients. Due to practical limitations, the sample size for lKTx patients (N = 26) in this study is smaller than the calculated one. Accordingly, the findings of the present study referring to the lKTx group should be interpreted with caution and considered exploratory.

## Results

### Patients’ Demographics

In total, 438 patients were included in the study, aged 54 (42–62) years. Out of them 56 were patients with CKD-V, 207 patients on HD for at least 1 year, 149 rKTx patients (1 year post-transplantation), and 26 lKTx patients (>17 years post-transplantation). The sex and median age of the patients did not differ among groups [58 (39–69) years for CKD-V, 55 (43–62) years for HD, 51 (41–57) years for rKTx and 58 (45–66) years for lKTx patients, p = 0.788] The control group included 49 healthy individuals aged 53 (39–64) years. Patients’ demographics are demonstrated in Table 1. In the rKTx group, 22 patients had received ATG as an induction treatment, as described above. Moreover, seven patients had a rejection episode during the first year, 5 out of which had biopsy-proven T cell mediated rejection and received ATG for treatment. All these patients had received ATG for induction. The rest two patients had antibody mediated rejection and were treated with plasma exchange, corticosteroids and intravenous immunoglobulin. In the lKTx group, one patient had a T cell mediated rejection episode and he was treated with ATG both for induction and rejection. Another one patient had an antibody mediated rejection and was also treated with corticosteroids and plasma exchange. Moreover, in the same group, two patients were not on calcineurin inhibitors at the time of flow cytometry analysis, for three and 5 years, because of chronic toxicity. Moreover, three of the patients in the rKTx cohort had a PCR proven CMV reactivation. All of these patients were already CMV seropositive at transplantation.

**TABLE 1:** Patients’ demographics.

Patients’ characteristics		CKD-V	HD	rKTx	lKTx
Patients	Ν	56	207	149	26
Median age, years		58 (39–69)	55 (43–62)	51 (41–57)	58 (45–66)
Sex, male/female		32/24	123/84	99/50	12/14
*Cause of CKD*					
Primary glomerulonephritis		11	49	42	11
Hypertension		16	33	26	5
Diabetes mellitus		16	28	11	3
Polycystic kidney disease		5	21	27	4
Obstructive nephropathy		0	14	18	1
Other		0	12	11	0
Unknown		8	50	14	2
*Treatment modality*					
Online hemodiafiltration		​	54	​	​
Median dialysis vintage		​	84 (19–126)	84 (24–126)	30 (16–65)
*Comorbidities*					
Hypertension		50	143	115	24
Cardiovascular disease		11	54	45	18
Diabetes mellitus		16	30	27	7
Dyslipidemia		21	77	64	20
History of kidney transplantation (%)		-	35	-	-
ATG treatment, N (%)		​	​	22 (14.7)	5 (19.2)
Preemptive Ktx		​	​	12	3
Live donor		​	​	44	16
Delayed graft function		​	​	22	2
Rejection episode		​	​	7	2
Retransplantation (%)		​	​	13 (8.7)	4 (15.3)
Pre-transplantation anti-HLA sensitization (PRA >5%)		​	​	56	11

Continuous variables are reported as median (25th – 75th percentile), CKD-V: chronic kidney disease stage V, HD: hemodialysis, rKTx: recent kidney transplantation, lKTx: long-term kidney transplantation.

### Differences in Lymphocytic Subpopulations Among Patients

**FIGURE 2:**
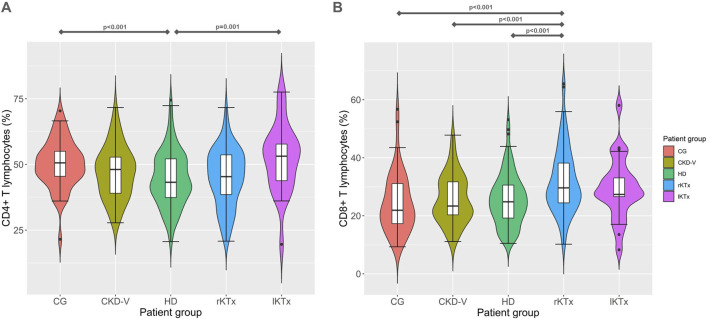
**(A)** CD4^+^ and **(B)** CD8^+^ T cell proportion kinetics in different CKD stages. CG: control group, CKD-V: chronic kidney disease stage V, HD: hemodialysis, rKTx: recent kidney transplantation, lKTx: long-term kidney transplantation. The arrows indicate groups with statistically significant differences between the examined parameters, after Bonferronni correction.

Table 2 shows the differences of lymphocytic cell subsets in patients’ groups, compared to the CG. In the following comparisons of lymphocyte subpopulations, the level of statistical significance was set at p < 0.005, after Bonferroni correction. Total lymphocyte percentage was reduced in patients with CKD-V compared to CG, 20.2 (14.4–25.8) vs. 29.7 (24.4–38.1)%, respectively, p < 0.001, without further substantial deterioration after dialysis initiation [19.9 (15.3–23.7)% in HD, p = 0.83 compared to CKD-V]. Kidney transplantation resulted in an early increase in total lymphocyte percentage during the first year, 24.7 (20–32.6) vs. 19.9 (15.3–23.7)% in rKTx and HD respectively, p < 0.001, with no increase lKTx 25.5 (21.9–35)%, p = 0.27 compared to rKTx and p = 0.16 compared to CG (Table 2). Similar kinetics were observed in CD4^+^ subpopulation proportion (Figure 2A), however, with CD8^+^ T lymphocytes percentage presenting a gradual upward tendency which was significant in the rKTx group compared to CG, CKD-V and HD [29.6 (24.3–38.3) in rKTx vs. 21.9 (17.1–31.1)% in CG, p < 0.001, vs. 23.3 (20.1–31.9)% in CKD-V p < 0.001, vs. 24.8 (19.1–30.6)% in HD, p < 0.001, (Figure 2B). Similar kinetics were observed in the total counts of total lymphocytes and CD4^+^ T cells, whereas the total count of CD8^+^ T cells was reduced in HD in comparison to HC and peaked in the lKTx group (Supplementary Table S1; Supplementary Figure S1).

**TABLE 2:** Differences in proportions of lymphocytic subpopulations among healthy controls and patient groups of different stage of chronic kidney disease.

Cell population, %	CG	CKD-V	KD	rKTx	lKTx	p
​	Ν = 49	Ν = 56	Ν = 207	Ν = 149	Ν = 26	​
Total lymphocytes	29.7 (24.4–38.1)	20.2 (14.4–25.8)^a^	19.9 (15.3–23.7)^a^	24.7 (20–32.6)^a^ ^,^ ^b^ ^,^ ^c^	25.5 (21.9–35)^b^ ^,^ ^c^	<0.001
CD4^+^	50.6 (45.1–55.1)	48.1 (38.7–52.8)	43.2 (37.4–52.1)^a^	45.3 (38.1–53.8)	53.1 (43.1–59)^c^	<0.001
CD8^+^	21.9 (17.1–31.1)	23.3 (20.1–31.9)	24.8 (19.1–30.6)	29.6 (24.3–38.3)^a^ ^,^ ^b^ ^,^ ^c^	27.3 (24.7–34.4)	<0.001
NK	10.9 (9.4–15.8)	12.2 (9.9–19.8)	17.6 (11.8–24.3)	8.9 (5.6–14.7)^c^	9.5 (5.1–14.3)^c^	<0.001
Tregs	5.9 (4.4–7.4)	6 (3.7–7.9)	4.4 (2.9–5.9)^a^ ^,^ ^b^	4.2 (3.2–5.3)^a^ ^,^ ^b^	2.8 (1.7–3.7)^a^ ^,^ ^b^ ^,^ ^c^ ^,^ ^d^	<0.001
CD4^+^CD28^−^	3.5 (1.4–6,8)	3.8 (1–11.9)	6 (2.1–13.2)^a^	4.6 (1.5–11.1)	12.6 (4.7–27.6)^a^ ^,^ ^b^ ^,^ ^c^ ^,^ ^d^	<0.001
CD8^+^CD28^−^	38 (24.4–49.8)	49.3 (24.2–63.7)^a^	45.5 (28.4–58.9)^a^	39.1 (26.7–59.9)	68.4 (54.4–90.3)^a^ ^,^ ^b^ ^,^ ^c^ ^,^ ^d^	<0.001

CG: control group, CKD-V: chronic kidney disease stage V, HD: hemodialysis, rKTx: recent kidney transplantation, lKTx: long-term kidney transplantation.

^a^ Statistical significance from CG.

^b^ Statistical significance from CKD-V.

^c^ Statistical significance from HD.

^d^ Statistical significance from rKTx. For the above comparisons statistical significance level has been set to <0.005 after Bonferroni correction.

**FIGURE 3:**
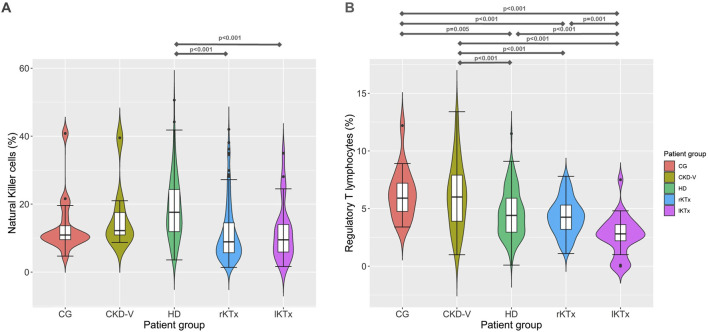
**(A)** Νatural killer cells and **(B)** regulatory T cell proportion kinetics in different CKD stages. CG: control group, CKD-V: chronic kidney disease stage V, HD: hemodialysis, rKTx: recent kidney transplantation, lKTx: long-term kidney transplantation. The arrows indicate groups with statistically significant differences between the examined parameters, after Bonferronni correction.

HD patients presented a significant increase of NK proportion compared to CG [17.6 (11.8–24.3) vs. 10.9 (9.4–15.8)% respectively, p = 0.009]. In the rKTx group, NK were found to have significantly decreased in comparison to HD [8.9 (5.6–14.9) vs. 17.6 (11.8–24.3)%, p < 0.001], without difference from CG (p = 0.07) and lKTX patients (p = 0.70) (Figure 3A). The count of NK cells was found to reach a nadir in the group of rKTx, but the fluctuations were not significant after Bonferroni correction (Supplementary Table S1; Supplementary Figure S2).

Treg percentage was found to be within normal range in CKD-V patients [6 (3.7–7.9) vs. 5.9 (4.4–7.4)%, for CKD-V and CG, respectively, p = 0.76] but showed a decline after HD initiation, 4.4 (2.9–5.9)%, p < 0.001 compared to CKD-V. One year after kidney transplantation, Treg percentage remained stable [4.2 (3.2–5.3)%, p = 0.30, in comparison to HD, but a further decline was observed in long-term transplantation 2.8 (1.7–3.7)%, p < 0.001 in comparison to rKTx (Figure 3B). The Treg count decreased gradually from CG to HD, following the total lymphocyte decrease. However, a slight but significant increase was observed in Treg count after transplantation, despite the drop in their proportion (Supplementary Table S1; Supplementary Figure S2).

**FIGURE 4:**
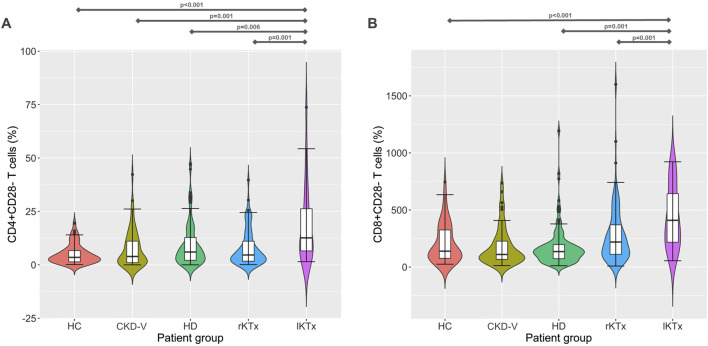
**(A)** CD4^+^CD28^−^ and **(B)** CD8^+^CD28^−^ T cell proportion kinetics in different CKD stages. CG: control group, CKD-V: chronic kidney disease stage V, HD: hemodialysis, rKTx: recent kidney transplantation, lKTx: long-term kidney transplantation. The arrows indicate groups with statistically significant differences between the examined parameters, after Bonferronni correction.

The percentage of CD28^−^ T cell subpopulations increased in HD patients compared to CG, with CD8^+^CD28^−^ increasing already before dialysis initiation, in the latest stages of CKD-V, 38 (24.4–49.8), 49.3 (24.2–63.7) and 45.5 (28.4–58.9)% for CG, CKD-V and HD respectively, p = 0.049 for CKD-V vs. CG and p = 0.026, for HD vs. CG. In the CD4^+^CD28^−^ subpopulation, the increase was observed only after HD initiation, 3.5 (1.4–6.8), 3.8 (1–11.9) and 6 (2.1–13.2)%, for CG, CKD-V and HD respectively, p = 0.64 for CKD-V vs. CG and p = 0.02 for CKD-V vs. HD. However, the above changes were not significant after Bonferonni correction. Kidney transplantation resulted in restoration of CD4^+^CD28^−^ and CD8^+^CD28^−^ to normal levels already from the first year after transplantation. However, in the group of long-term transplanted patients, an increase in both CD28^‐^ subpopulations was observed to levels considerably higher even than from those of HD patients, CD4^+^CD28^‐^: 12.6 (4.7–27.6) vs. 6 (2.1–13.2)%, for lKTx and HD respectively, p = 0.006, CD8^+^CD28^‐^: 68.4 (54.4–90.3) vs. 45.5 (28.4–58.9)% for lKTx and HD respectively, p < 0.001 (Figure 4). The steep increase of CD28^−^ T cell subsets in lKTx patients was also significant when total cell counts were examined (Supplementary Table S1).

### Patients Sex, Age, Previous CMV Infection and Renal Function Were the Main Parameters Associated With the Immune Phenotype

Immune phenotype differed significantly between male and female patients in the HD group. Women had an increased total lymphocyte proportion in comparison to men [21.4 (17.5–25.9) vs. 18.1 (14.6–22.4)%, p < 0.001] which was due to increased CD4^+^ T cell proportion [46.5 (38.1–53.8) vs. 42.3 (36–49)%, p = 0.038], but not CD8^+^ T cells [24.3 (20.3–29.2) vs. 25.3 (18.1–31.6)%, p = 0.99)]. Moreover, NK were decreased in women on HD [14.5 (10.3–20.7) vs. 19.3 (14.4–26.6)%, p < 0.001], with no differences in the rest of analyzed cell subsets. Sex remained significant after inclusion of age and CMV status in a multivariate model only for total lymphocytes percentage (95% CI: 0.025–0.321, p = 0.023) and NK (95% CI: -0.587 – -0.071, p = 0.013) but not for CD4^+^ T cells percentage (95% CI: -0.034 – 0.182, p = 0.178). In comparison, in the CG, only CD4^+^ T cell proportion differed between male and female patients [46.2 (39.2–53.6) vs. 43.9 (37.5–47.9)% for females and males respectively, p = 0.028].

**FIGURE 5:**
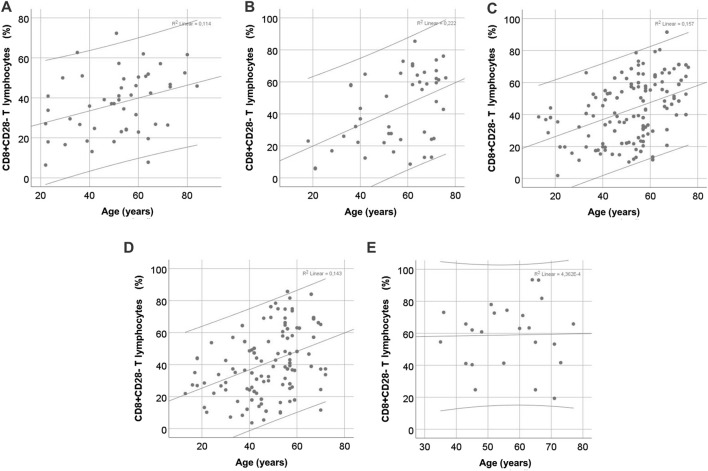
Correlation of CD8^+^CD28^−^ T cell proportion with age in different CKD stages. **(A)** control group, **(B)** chronic kidney disease stage V, **(C)** hemodialysis, **(D)** recent kidney transplantation, **(E)** long-term kidney transplantation.

Patients’ age was a significant predictor of the percentage of CD8^+^CD28^−^ T cells in all patient groups studied, as well as in healthy volunteers, except for the lKTx group (Figure 5). The linear regression data are shown in Table 3. In kidney transplant patients, both rKTx and lKTx, age was an independent predictor of the percentage of CD4^+^CD28^−^ T cells (Table 3). In the rKTx group, age was an independent predictor for several T cell populations, such as total lymphocytes percentage (r = −0.31, R^2^ = 0.097, 95% CI: −0.275 – −0.088, p < 0.001), NK percentage (r = 0.26, R^2^ = 0.071, 95% CI: 0.066–0.276, p = 0.002) CD4^+^ T lymphocytes count (r = −0.4, R^2^ = 0.16, 95% CI: −15.34 – −6.92, p < 0.001) and Tregs count (r = −0.31, R^2^ = 0.09, 95% CI: −0.685 – −0.216, p < 0.001). In the lKTx group, NK proportion depended also on the age (r = 0.49, R^2^ = 0.21, 95% CI: 0.073–0.643, p = 0.016).

**TABLE 3:** Uni- and multivariate linear regression for predictions of CD4^+^CD28^−^ and CD8^+^CD28^−^ T cell percentage, based on age, in patient groups of different stages of chronic kidney disease.

Patient group	Univariate linear regression				Multivariate linear regression		
	B	R^2^	95% CI	*p*	B	95% CI	*p*
CD4^+^CD28^−^	​	​	​	​	​	​	​
CG	0.04	0.002	−0.018 – 0.024	0.791	–	–	–
CKD-V	0.31	0.1	0.002–0.048	0.036	0.56	−0.003 – 0.091	0.058
HD	0.09	0.008	−0.006 – 0.024	0.226	–	–	–
rKTx	0.30	0.09	0.015–0.046	<0.001	0.25	0.005–0.046	0.014
lKTx	0.47	0.22	0.009–0.083	0.018	0.48	0.007–0.085	0.024
​	​	​	​	​	​	​	​
CD8^+^CD28^−^	​	​	​	​	​	​	​
CG	0.33	0.11	0.002–0.02	0.02	–	–	–
CKD-V	0.48	0.23	0.009–0.029	0.001	0.68	−0.029 – 0.083	0.22
HD	0.37	0.14	0.01–0.02	<0.001	0.32	0.004–0.022	0.005
rKTx	0.33	0.11	0.008–0.022	<0.001	0.30	0.005–0.023	0.004
lKTx	0.04	0.002	−0.017 – 0.14	0.839	–	–	–

CG: control group, CKD-V: chronic kidney disease stage V, HD: hemodialysis, rKTx: recent kidney transplantation, lKTx: long-term kidney transplantation. In the multivariate linear regression models anti-CMV antibodies titres and eGFR were included.

Anti-CMV IgG antibodies levels could predict the percentage of CD4^+^CD28^−^ and CD8^+^CD28^−^ T lymphocytes in HD patients, (r = 0.29, R^2^ = 0.08, 95% CI: 0.001–0.007, p = 0.011 and r = 0.28, R^2^ = 0.08, 95% CI: 0.000–0.03, p = 0.015 respectively). However, in multivariate regression including age, the antibody levels did not remain statistically significant for the prediction of CD8^+^CD28^−^ T lymphocytes and age remained the only independent factor. Correlation of anti-CMV IgG antibody levels with the percentage of CD4^+^CD28^−^ T lymphocytes, (r = 0.31, R^2^ = 0.1, 95% CI: 0.002–0.007, p = 0.002) was also observed in rKTx patients but not in the lKTx group (r = 0.071, R^2^ = 0.1, 95% CI: -0.002 – 0.029, p = 0.072). In multivariate analysis, both antibody levels and age remained independent factors for the percentage of CD4^+^CD28^−^ T lymphocytes. Finally, eGFR was not a predictor of the percentage of CD28^−^ T lymphocytes in either of the groups of transplanted patients.

Dialysis vintage (DV) was associated negatively to the total lymphocytes proportion in HD patients (r = −0.18, R^2^ = 0.035, 95% CI: -0.002 – 0.000, p = 0.016), affecting mainly CD4^+^ T cells (r = −0.22, R^2^ = 0.052, 95% CI: −0.002 – 0.000, p = 0.003), but not CD8^+^ T cells (r = 0.06, R^2^ = 0.004, 95% CI: −0.001 – 0.001, p = 0.468). NK were positively associated to DV (r = 0.19, R^2^ = 0.038, 95% CI: 0.000–0.004, p = 0.017), while Tregs decreased as DV increased (r = −0.17, R^2^ = 0.028, 95% CI: -0.003 – 0.000, p = 0.031). CD4^+^CD28^−^ T cells proportion was also affected (r = 0.17, R^2^ = 0.03, 95% CI: 0.000–0.009, p = 0.035), but not CD8^+^CD28^−^ T cells proportion (r = −0.08, R^2^ = 0.007, 95% CI: -0.001 – 0.003, p = 0.297). However, in the multivariate models including sex, age and CMV status, DV was significant for none of the analyzed cell subsets. This effect of DV in total lymphocytes and CD4^+^ T cells was also retained during the first year of transplantation, but was not significant in multivariate analysis or lKTx patients.

Estimated GFR was a predictor of CD4^+^ and CD8^+^ T cell proportion (r = 0.21, R^2^ = 0.045, 95% CI: 0.001–0.005, p = 0.012 and r = 0.17, R^2^ = 0.029, 95% CI: −0.006 – 0.000, p = 0.043, respectively) and Tregs count (r = 0.21, R^2^ = 0.047, 95% CI: 0.002–0.012, p = 0.01) in rKTx patients, but this correlation did not remain significant in multivariate analysis including age and anti-CMV antibodies levels.

### ATG Treatment Affects Immune Phenotype

History of ATG treatment in the transplant patient groups had impact on phenotype of T cells. In the rKTx groups N = 22 patients had received ATG for induction therapy. These patients had a lower CD4^+^ T cell proportion in comparison to patients who had not received ATG [36.9 (29.6–45.9) vs. 47.7 (40.2–53.9)% respectively, p = 0.003], as well as more CD8^+^ T cells [38 (25.9–46.3) vs. 28.9 (24.1–36.8)% respectively, p = 0.037], and more CD8^+^CD28^−^ T cells [49 (37–69) vs. 37 (24–57)% respectively, p = 0.013] (Supplementary Table S2). However, in the multivariate linear model with age, sex and CMV status as confounding factors, ATG treatment significantly affected only the CD4^+^ T cell proportion (95% CI: -0.367 – -0.09, p = 0.002), along with age, but neither of CD8^+^ or CD8^+^CD28^−^ T cells, and age was a significant factor for CD8^+^CD28^−^ T cells percentage. In the lKTx group no difference was found in the immune phenotype according to ATG treatment (N = 5 patients had received ATG, data on Supplementary Table S2). In order to increase the generalizability of the study and eliminate the impact of ATG treatment on lymphocytic subsets, we performed the comparisons of the examined subsets among the patients groups, after excluding the ATG treated patients. The kinetics of subpopulations were not substantially altered, as shown in the Supplementary Table S3.

## Discussion

In this study, changes in specific lymphocyte subpopulations were examined across the spectrum of CKD, from the pre-end stage to long-term transplantation. One of the main conclusions was that CKD, especially in the end-stage, is associated with lymphopenia and a decrease in CD4^+^ T lymphocytes, perhaps with a disruption in the ratio between them. Moreover, successful kidney transplantation was found to restore total lymphocyte counts, as well as CD4^+^ to normal levels especially in the long term, with a gradual increase of CD8^+^ T lymphocyte proportion, up to the first-year post-transplantation. However, in the setting of lKTx no improvement in CD28^−^ T cell proportion was found, but rather a great increase.

Changes in CD4^+^ and CD8^+^ T lymphocyte populations in CKD have been described in several studies, without their findings being consistent [4]. In a study published in 2004, an increase in total lymphocytes and CD4^+^ T cell counts was described as early as 4 weeks after kidney transplantation [10]. However, the underlying mechanism is not well understood, as data on the recovery of thymic function after transplantation are insufficient [11, 12]. It is worth noting that in another more recent study, it was shown that CD4^+^ T cell counts were lower compared to patients with CKD or healthy subjects, despite an increase in total T cell counts, 3 years after transplantation [13]. Nonetheless, it is not clear whether this numerical amelioration is accompanied by functional restoration. Chronic exposure to the uremic milieu may deeply affect lymphocytic function, a change that has not been proven to reverse after transplantation. It is interesting that old CD4^+^ T cells have compromised metabolic pathways due to dysfunctional mitochondria accumulation and are not able to cover their energy demands after activation [14]. Moreover, their capacity to form immunological synapses with antigen-presenting cells is severely reduced [15].

The literature regarding alterations of Tregs after kidney transplantation remains very restricted. One study published in 2019 reports low levels of Tregs in the first 6 months after kidney transplantation, followed by a gradual recovery to near baseline levels during the first year [16]. In addition, the study by Wang et al. reported a further decrease in the number of Tregs after transplantation compared with CKD [13], but these findings were not confirmed by our study. It is likely that increased immunosuppression during the early period after transplantation plays a role in this phenomenon [17].

Reduced levels of Tregs likely have an important clinical role, as they have been associated with an increased risk of acute rejection and chronic graft dysfunction [18, 19]. Decreased proportions of Tregs have been reported in stable kidney graft recipients under immunosuppressive treatment [20]. However, the same study presents differences in the functional state of Tregs, highlighting the role of Foxp3 demethylation in these cells, which may be capable of inducing tolerance in neighboring effector T cells, reducing their reactivity [21]. This ability is impaired by the proinflammatory state in the setting of transplantation, potentially under the effect of interleukin (IL) 6 [21]. IL-6 can be secreted independently of CD28 costimulation, but in T cells, CD28 signaling acts as a major amplifier of IL-6 production [22]. Moreover, experimental inhibition of IL-6 resulted in increase in Tregs frequency and function, and prolonged graft survival [21]. Whether loss of CD28 expression is associated with an improvement in Tregs number and function remains to be clarified, but is supported by our data (Supplementary Figure S3).

Data on NK changes in CKD and after kidney transplantation are even more limited. In a study published in 2016, a low frequency of NK was associated with immune risk phenotype (IRP) in transplant recipients both before and 1 year post-transplantation [23]. Although no specific immune phenotype was associated with opportunistic infection, the latter was more frequent in patients with IRP. The relationship between NK phenotype and IRP in dialysis patients was also reported by Dewolfe et al. The authors associated NK frequence and distinctive phenotypic characteristics of NK with IRP + status in dialysis patients [24]. Moreover, another small study found no difference in NK cell proportions or counts between healthy women and those who had undergone transplantation [25]. Adequate NK function may be of great interest in the context of transplantation. Besides immunosurveillance, NK have been shown to eliminate senescent T cells *in vitro* and *in vivo* [26], which secrete pro-inflammatory mediators, resulting in low-level chronic inflammation, associated with tissue dysfunction. It is worth noting that senescent T cells do not originate only from the recipient, but also senescent T cell from the graft may participate to increased inflammatory activity [26].

Despite age being a significant factor affecting T cell subsets in healthy individuals [27], data on the role of age in immune phenotype of CKD patients remain inconclusive [4, 7]. However, it has been reported that after transplantation, only younger individuals were capable of restoring, at least partially, the proportion of naïve T cells [28]. Moreover, expansion of the terminally differentiated T cell subset, expressing CD57 epitope, has been found to elderly kidney transplant recipients 1 year after transplantation and was associated with CMV infection [29]. This “memory expansion” of terminally differentiated cells occurs already during the first year after transplantation, and may not be related to clinical infection or viremia [30]. These cells are deemed senescent as their express several senescence features, among other telomere attrition [4, 29]. Our findings regarding age-dependent acceleration of senescence in recent transplant recipients are not discordant to these previous findings, as expression of CD57 interrelates to loss of CD28 expression, and the resulting cells are considered highly senescent [4, 31]. However, Schaenman et al. found no significant difference in CD28^−^ T cell percentage between young and elder kidney graft recipients [28].

One of the most interesting findings of our study was the prominent increase in CD28^−^ T cell subsets in kidney graft recipients after long-term transplantation, which was found to be independent of age. Similar results were reported in another study, in kidney grafts recipients, at least 4 years post-transplantation [32], with CD8^+^CD28^−^ T cells expressing innate-like receptors [9]. However, it is unclear whether CD28^−^ T cell subset expansion, due to other reasons, leads to increased graft survival, or transplantation *per se* results in accumulation of CD28^−^ T cells. We are inclined to believe for the latter mechanism for several reasons. Firstly, CD28^−^ T cell proportion showed a downward tendency during the first year after transplantation, and in any case their proportion did not increase. Secondly, principal mechanism of CD28 loss is the repeated activation of the memory cell, leading to downregulation of transcription factor for CD28 [33], thus we can hypothesize that the longer the antigenic stimulation, the more prominent the CD28 loss [34]. Tolerance to the graft has been associated with the recipient age, with younger recipients having a greater risk to develop *de novo* Donor Specific Antibodies than elder patients [35]. Moreover, upregulation of CD28 expression in older individuals resulted in acceleration of graft rejection, in experimental animal models [36]. However, in our study, age was not found to be a confounding factor for CD8^+^CD28^−^ T cells proportion.

Transplant therapy regimen was found to affect immune phenotype in the rKTx group. ATG has been shown to induce apoptosis in CD4^+^CD28^−^ T cells, a T cell subset known for apoptosis deficits, rather than in CD4^+^CD28^+^ T cells, shortly after administration, in a small study published in 2012 [37]. However, levels of this T cell subset did not differ between patients with or without ATG administration, 1 year after transplantation. Of note, it has been postulated that one mechanism of increase of highly differentiated cell subsets, such as CD28^−^, is the rapid cell proliferation after cell elimination [38]. The same mechanism is also proposed by Lee et al. who associated ATG treatment with decreased CD4^+^ and increased CD28^−^ T cells, without reporting any correlation with patients’ age. This latter study also suggests severe functional deficits in lymphocytes of ATG treated patients, that may last for up to 1 year after transplantation [39]. Nonetheless, according to our findings, CD28^−^ T cell expansion appears to be independent of treatment regimen in lKtx recipients. Similar results were reported by another study, for CMV responsive memory cells, which is a subset that tends to expand over time [30, 40].

Other immunosuppressive agents, widely used in transplantation, may have impact on CD28^−^ T cell subsets. It has been reported that kidney graft recipients have increased proportions of CD28^−^ T cells, even in the absence of CMV infection [41]. Even though these cells are deemed to be prone in apoptosis, their survival may increase in the presence of IL-6 and 15 [21, 42–44]. Il-15 may also promote CD28 expression loss *in vitro* [45]. Increased secretion of IL-6 may begin shortly after transplantation due to ischemia-reperfusion injury, innate immune system activation, and alloreactions against class II HLA molecules [21]. Whether the persistence of these reactions for years can lead to CD28^−^ T cell expansion is not clear. Moreover, CD28 signaling is key to IL-2 production, which is reported to be low in KTx patients [41, 46]. Treatment with tacrolimus has been associated with low IL-2 secretion in old CD4^+^ but not CD8^+^ T cells [47]. Moreover, cyclosporine A was reported to reduce telomerase activity, in T cell, *in vivo* [32]. Even though CD28^−^ T cells are characterized by reduced telomerase activity, it is not clear whether cyclosporine A could also decrease CD28 expression.

We acknowledge several limitations in our study. Even though the examined populations were age and sex matched, we could not include comparable number of individuals in each patient group, and as a result some populations, such as lKTx patients, are under-represented. Moreover, this is a single center study and therefore, the generalizability of the results may be limited. The design of the study also reduces reliability of the results, as a longitudinal examination of the same patients at different timepoints would offer a better insight in the immune alterations. Despite the numerical changes in the NK cells found, no data on intensity of CD56 expression were available, as the reagent used was a combined CD16‐CD56 antigen. Dim or bright expression of CD56 has been associated with functional differences. Finally, this study did not demonstrate any potential molecular mechanisms leading to these observations, nor associated the findings with clinical characteristics of the patients, such as rejection episodes or opportunistic infections. Especially for the lKTx cohort, CD28^−^ T cell expansion may predispose to cardiovascular and infectious complications [48].

Overall, our results indicate a significant fluctuation of the phenotype of cellular immunity components across the range of CKD. Receiving a kidney graft is capable of restoring some of these alterations shortly after transplantation, however the long-term antigenic stimulation in lKTx patients is associated with senescent T cell subsets expansion.

## Data Availability

The raw data supporting the conclusions of this article will be made available by the authors, without undue reservation.
