# Supplementary figures and images for: Cellular Immunity in Chronic Kidney Disease and Changes After Kidney Transplantation
Source: Transpl Int. 2026 Feb 3;39:15622. doi: 10.3389/ti.2026.15622 (PMC12862250; doi:10.3389/ti.2026.15622)

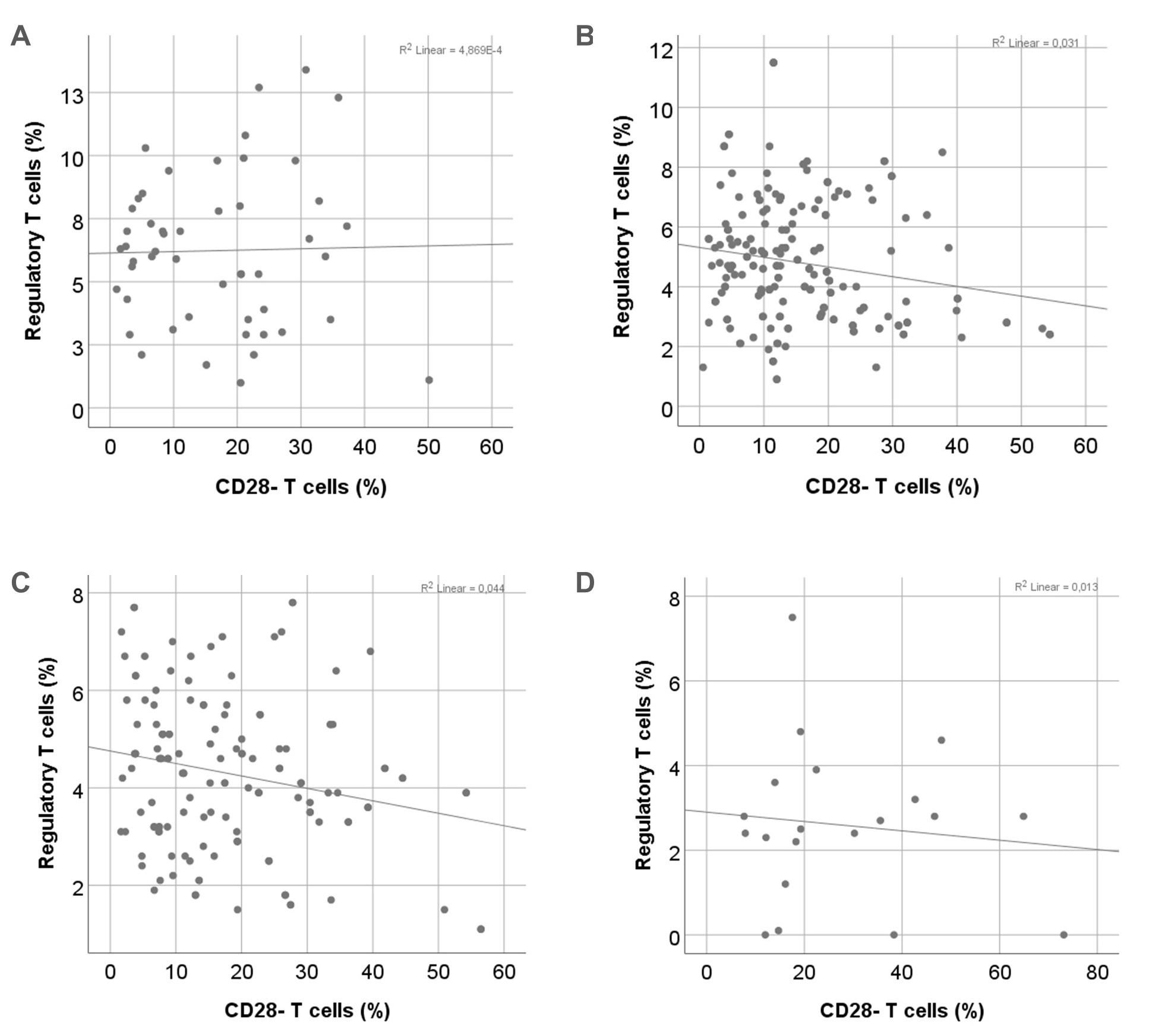

Supplement: Supplementary file 1 [file Image3.jpeg]

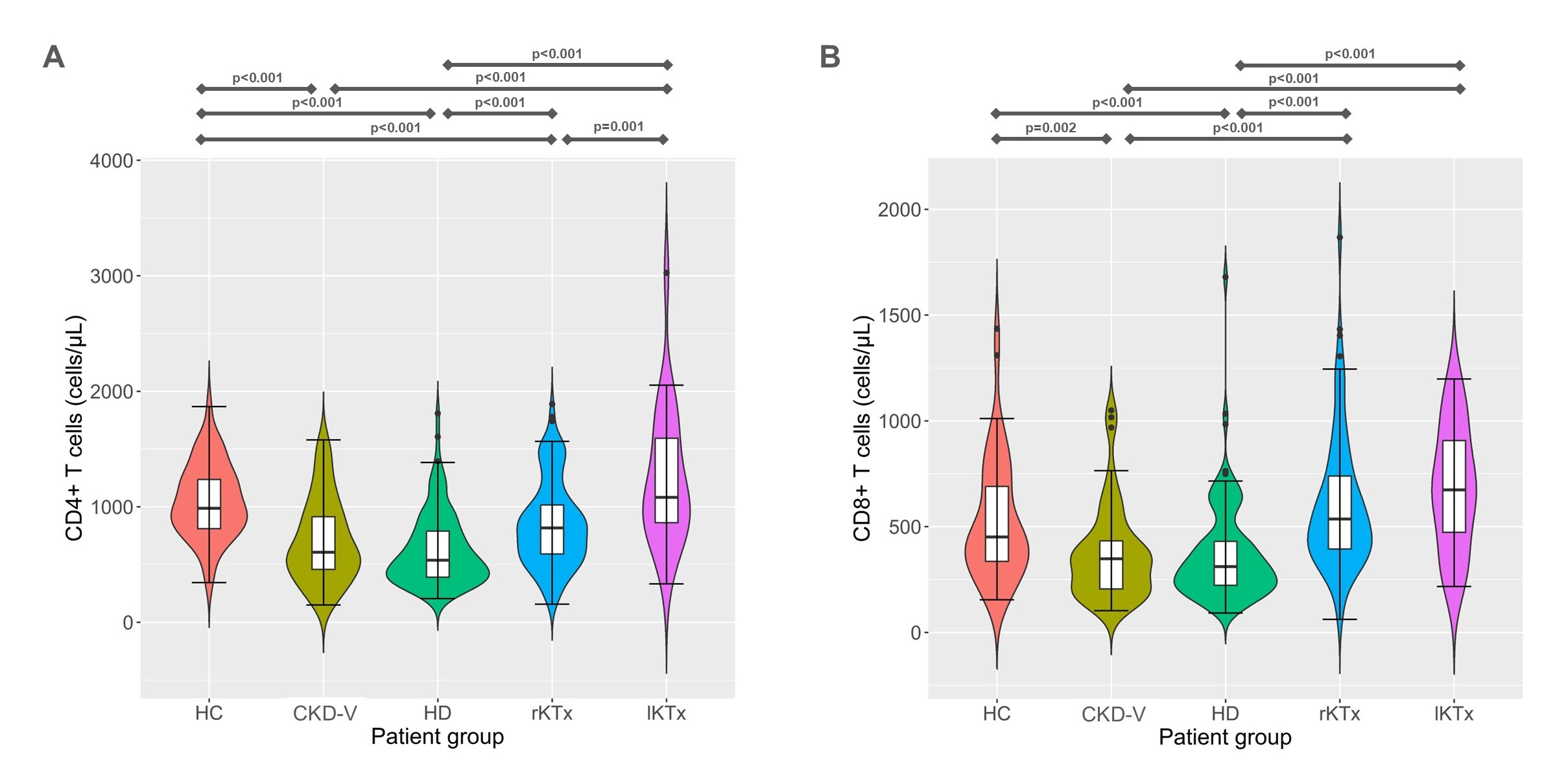

Supplement: Supplementary file 3 [file Image1.jpeg]

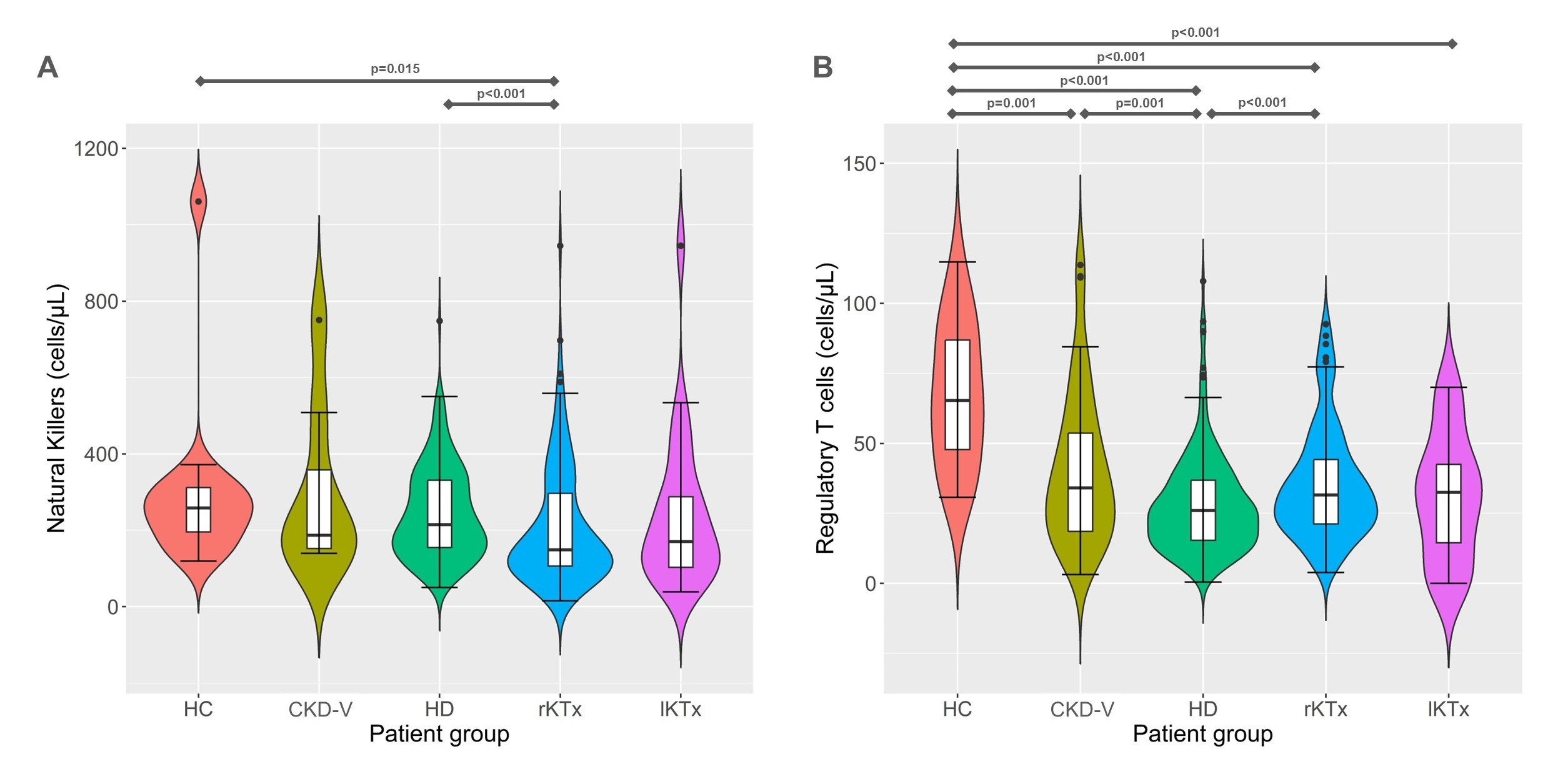

Supplement: Supplementary file 4 [file Image2.jpeg]
