# Supplementary material for: Cellular Immunity in Chronic Kidney Disease and Changes After Kidney Transplantation
Source: Transpl Int. 2026 Feb 3;39:15622. doi: 10.3389/ti.2026.15622 (PMC12862250; doi:10.3389/ti.2026.15622)
Supplement: Supplementary file 2 [file Table1.docx]

**Supplement**

**Capsule Sentence Summary**

Kidney transplantation may restore total lymphocytes counts, already during the first year. However, long term graft survival comes at a cost of increased senescent CD28- T cells, independently of age.

**Supplemental Table 1.** Differences in counts of lymphocytic subpopulations among healthy controls and patient groups of different stage of chronic kidney disease. CG: control group, CKD-V: chronic kidney disease stage V, HD: hemodialysis, rKTx: recent kidney transplantation, lKTx: long-term kidney transplantation, *statistical significance from CG, + statistical significance from CKD-V, # statistical significance from HD, $ statistical significance from rKTx. For the above comparisons statistical significance level has been set to <0.005 after Bonferroni correction

|  | **CG** | **CKD-V** | **HD** | **rKTx** | **lKTx** | **p** |
| --- | --- | --- | --- | --- | --- | --- |
| **Cell population (cells/μl)** | Ν=49 | Ν=56 | Ν=207 | Ν=149 | Ν=26 |  |
| Total lymphocytes | 2100 (1600-2500) | 1400 (1000-1700)***** | 1200 (1000-1700)***** | 1700 (1400-2300)***^+#^** | 2300 (1600-2700)**^+#$^** | <0.001 |
| CD4+ | 985 (803-1237) | 606 (441-925)***** | 536 (390-792)***** | 815 (589-1020)***^+#^** | 1080 (832-1639)**^+#$^** | <0.001 |
| CD8+ | 451 (335-717) | 347 (197-446)***** | 310 221-432)***** | 535 (394-739)**^+#^** | 674 (427-949)***^+#^** | <0.001 |
| NK | 258 (185-312) | 186 (152-458) | 214 154-331) | 148 (105-297)***^#^** | 170 (92-301) | 0.001 |
| Tregs | 65 (45-91) | 34 (18-55)***** | 25 (15-36)***^+^** | 31 (21-44)***^#^** | 32 (9-45)***** | <0.001 |
| CD4+CD28- | 37(14-76) | 24(6-51) | 28(13-70) | 38(15-86) | 124(48-256)***^+#$^** | <0.001 |
| CD8+CD28- | 138(75-332) | 109(64-229) | 135(72-198) | 218(111-371)**^+#^** | 409(208-687)***^+#$^** | <0.001 |

**Supplemental Table 2.** Differences in proportions of lymphocytic subpopulations between patients treated with ATG for induction therapy versus patients treated with Basiliximab

|  | **Recently transplanted patients** | | | **Long-term transplanted patients** | | |
| --- | --- | --- | --- | --- | --- | --- |
| **Cell population (%)** | **ATG** Ν=22 | **Basiliximab** Ν=147 | **p** | **ATG**  Ν=5 | **Basiliximab** Ν=21 | **p** |
| Total lymphocytes | 22.1(17.7-28) | 25.1(10.6-32.7) | 0.09 | 24.5(18.7-35.6) | 26.5(22.6-34.7) | 0.95 |
| CD4+ | 36.9(29.6-45.9 | 47.7(40.2-53.9) | 0.003 | 56.5(33.8-63.5) | 53(42.7-59.7) | 0.921 |
| CD8+ | 38(25.9-46.3) | 28.9(24.1-36.8) | 0.037 | 26.8(18.2-50.7) | 28.1(26.6-32.9) | 0.98 |
| NK | 9.3(6.2-21.1) | 8.7(5.2-13.9) | 0.294 | 12.4(7.2-14) | 8.4(4.6-16.8) | 0.745 |
| Tregs | 4.5(3.8-5.5) | 4.2(3.1-5.1) | 0.423 | 2.4(0.6-3.7) | 2.8(2.2-3.7) | 0.488 |
| CD4+CD28- | 6.6(2.7-19.7) | 4.4(1.4-9.9) | 0.165 | 19(4.7-45) | 12.3(3.8-25.6) | 0.629 |
| CD8+CD28- | 48.7(37.1-68.8) | 37.3(24.2-57) | 0.013 | 71(60-73) | 60.8(41-73) | 0.160 |

**Supplemental Table 3**. Differences in proportions of lymphocytic subpopulations among healthy controls and patient groups of different stage of chronic kidney disease **after excluding patients treated with ATG**. CG: control group, CKD-V: chronic kidney disease stage V, HD: hemodialysis, rKTx: recent kidney transplantation, lKTx: long-term kidney transplantation, *statistical significance from CG, + statistical significance from CKD-V, # statistical significance from HD, $ statistical significance from rKTx. For the above comparisons statistical significance level has been set to <0.005 after Bonferroni correction

|  | **CG** | **CKD-V** | **HD** | **rKTx** | **lKTx** | **p** |
| --- | --- | --- | --- | --- | --- | --- |
| **Cell population (cells/μl)** | Ν=49 | Ν=56 | Ν=207 | **Ν=127** | **Ν=21** |  |
| Total lymphocytes | 29.7(24.4-38.1) | 20.2(14.4-25.8)* | 19.9(15.3-23.7)* | 25.1(10.6-32.7)***^+#^** | 26.5(22.6-34.7)**^+#^** | <0.001 |
| CD4+ | 50.6(45.1-55.1) | 48.1(38.7-52.8) | 43.2(37.4-52.1)* | 47.7(40.2-53.9) | 53(42.7-59.7)^#^ | <0.001 |
| CD8+ | 21.9(17.1-31.1) | 23.3(20.1-31.9) | 24.8(19.1-30.6) | 28.9(24.1-36.8)***^+#^** | 28.1(26.6-32.9) | <0.001 |
| NK | 10.9(9.4-15.8) | 12.2(9.9-19.8) | 17.6(11.8-24.3) | 8.7(5.2-13.9)^#^ | 8.4(4.6-16.8)^#^ | <0.001 |
| Tregs | 5.9(4.4-7.4) | 6(3.7-7.9) | 4.4(2.9-5.9)***^+^** | 4.2(3.1-5.1)***^+^** | 2.8(2.2-3.7)***^+#$^** | <0.001 |
| CD4+CD28- | 3.5 (1.4-6,8) | 3.8 (1-11.9) | 6 (2.1-13.2)***** | 4.4(1.4-9.9) | 12.3(3.8-25.6)*^$^ | 0.006 |
| CD8+CD28- | 38 (24.4-49.8) | 49.3 (24.2-63.7)***** | 45.5 (28.4-58.9)***** | 37.3(24.2-57) | 60.8(41-73)*^$^ | 0.005 |


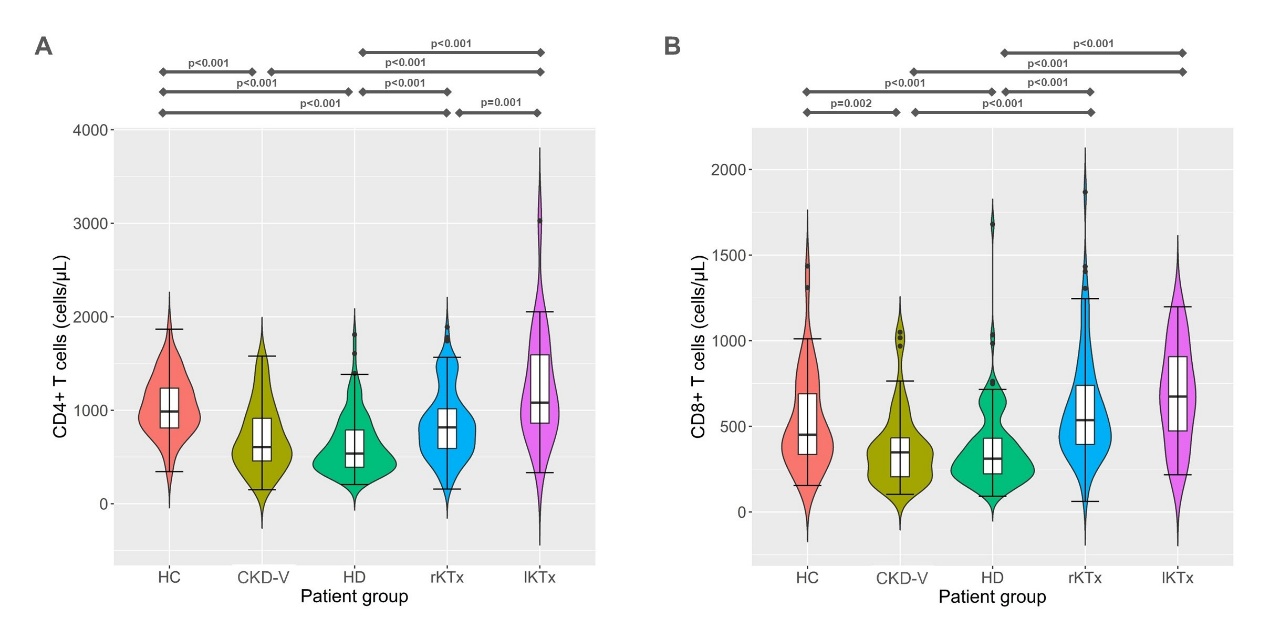


Supplemental Figure 1. (A) CD4+ and (B) CD8+ T cell count kinetics in different CKD stages. CG: control group, CKD-V: chronic kidney disease stage V, HD: hemodialysis, rKTx: recent kidney transplantation, lKTx: long-term kidney transplantation. The arrows indicate groups with statistically significant differences between the examined parameters


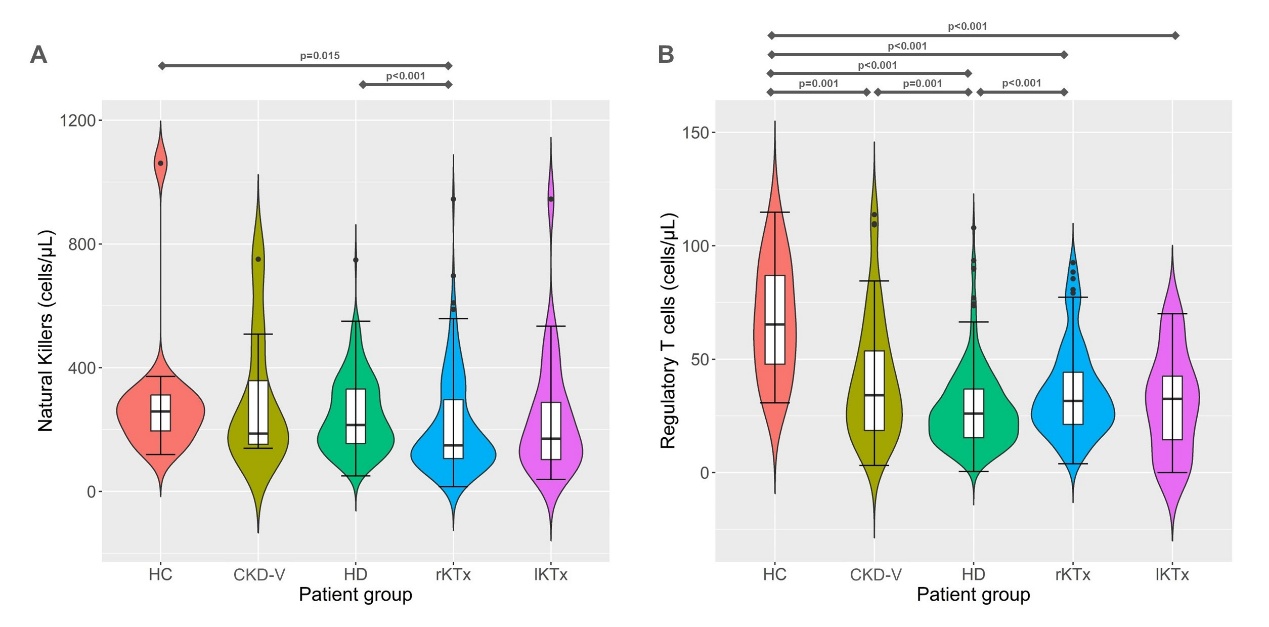


Supplemental Figure 2. (A) Νatural Κiller cells and (B) regulatory T cell count kinetics in different CKD stages. CG: control group, CKD-V: chronic kidney disease stage V, HD: hemodialysis, rKTx: recent kidney transplantation, lKTx: long-term kidney transplantation. The arrows indicate groups with statistically significant differences between the examined parameters.


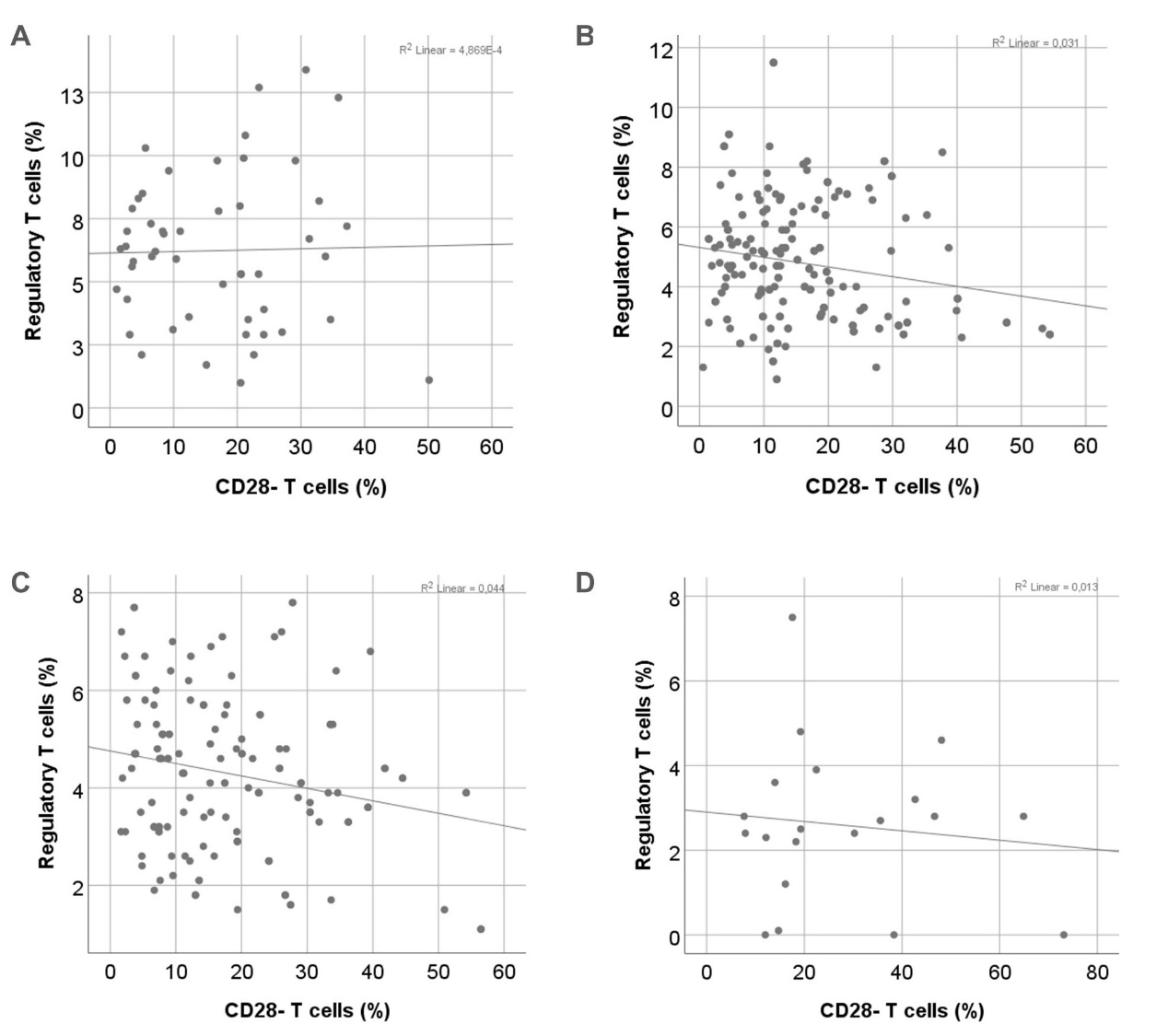


Supplemental Figure 3. Correlation of total CD28- with regulatory T cell proportions in different stages of CKD. A. CKD-V, r=0.002, p=0.987, B. HD, r=-0.165, p=0.024, C. rKTx, r=-0.17.4, p=0.042, D. lKTx, r=0.12, p=0.601.
